# Surgical Safety Checklist Implementation in a Tunisian University Hospital: A Mixed‐Methods Study of Compliance and Nurses’ Perspectives on Barriers, Facilitators, and Sustainability

**DOI:** 10.1155/jonm/7712576

**Published:** 2026-10-20

**Authors:** Wiem Aouicha, Mohamed Ayoub Tlili, Bushra Alshammari, Hamdi Lamine, Nojoud Abdullah Alrashidi, Nahed Mousa Saber, Amany Ali Abd Elsalam, Laila A. Hamed, Nick Sevdalis, Nadia Falah Shatti Al Enezi, Hind Mobark Fahd Alshamry, Maha Sanat Alrashedi, Aziza Zakaria Faramawy Ali

**Affiliations:** ^1^ Nursing Administration Department College of Nursing University of Hail Hail Saudi Arabia uoh.edu.sa; ^2^ Medical Surgical Nursing Department College of Nursing University of Hail Hail 21424 Saudi Arabia uoh.edu.sa; ^3^ Community Health Nursing Department College of Nursing University of Hail Hail Saudi Arabia uoh.edu.sa; ^4^ Maternal and Child Health Nursing Department College of Nursing University of Hail Hail Saudi Arabia uoh.edu.sa; ^5^ Medical-Surgical Nursing Department Faculty of Nursing Zagazig University Zagazig Egypt zu.edu.eg; ^6^ Centre for Behavioral and Implementation Science Interventions Yong Loo Lin School of Medicine National University of Singapore Singapore nus.edu.sg; ^7^ Nursing Administration Department Faculty of Nursing Benha University Benha Egypt bu.edu.eg

**Keywords:** barriers and facilitators, compliance, mixed-methods research, nursing leadership, patient safety, perioperative nursing, surgical safety checklist

## Abstract

**Background:**

The surgical safety checklist (SSC) is a valuable tool for promoting patient safety, but its implementation is complex and understudied, particularly in low‐ and middle‐income countries. Perioperative nurses are critical to the SSC’s successful implementation, but their experiences and the obstacles they encounter are underexplored under specific organizational systems.

**Objective:**

This study assessed SSC compliance and completion and investigated perioperative nurses’ perspectives and experiences using it in a Tunisian university hospital.

**Methods:**

A sequential mixed‐methods study was conducted over a 3‐month period. For the quantitative part, a retrospective review of 760 patient medical records was performed over a 19‐month period. Compliance and completion rates were analyzed and compared across all checklist phases and four surgical specialties. As for qualitative data, 13 semistructured interviews were conducted with perioperative nurses and then analyzed thematically to explore perceptions and experiences with the SSC. Participation was voluntary. Anonymity and confidentiality were ensured.

**Results:**

Quantitative findings revealed an SSC compliance proportion of 39.7%, which peaked at 60% during the implementation phase but declined to 29.4% during follow‐up. Compliance was significantly lower during night shifts and in emergency cases (*p* < 0.001). The “Sign‐Out” section was the most frequently omitted. Qualitative analysis identified five key themes: initial skepticism shifting to acceptance, barriers including time pressure and lack of accountability, facilitators such as leadership engagement and nursing champions, perceived positive impacts on teamwork, and concerns about long‐term sustainability.

**Conclusions:**

While the SSC is valued by nurses, its successful implementation and sustainability are critically dependent on the organizational culture within the operating room. Sustainable implementation requires formally integrating the SSC into hospital policy. This must be supported by strong leadership endorsement and strategic empowerment of nurse champions to overcome hierarchical barriers and foster a shared responsibility for patient safety.

**Implications for Nursing Management:**

Beyond policy and leadership commitment, sustaining SSC adherence requires psychological safety that enables staff to challenge unsafe practice, regular audit‐and‐feedback cycles to monitor compliance by specialty and shift, staffing and workflow that support SSC completion during high‐pressure periods, ongoing training, and clearly assigned responsibility for SSC use at the unit level.

## 1. Background

Surgical care is an essential component of global health, with over 313 million procedures performed annually worldwide [1]. Surgery can be lifesaving, but it also carries significant risk, with an estimated seven million patients experiencing surgical complications each year, and at least one million dying during or shortly after surgery [2]. The operating room (OR) is a complex, high‐risk environment that requires effective teamwork among surgeons, anesthetists, and perioperative nurses [3, 4]. Notably, a high percentage of adverse events is associated with surgery, accounting for nearly 40% of in‐hospital complications, with over a third considered preventable [5, 6].

In response, the World Health Organization (WHO) launched the *Safe Surgery Saves Lives* campaign, introducing the surgical safety checklist (SSC) as a simple yet powerful tool to decrease surgical complications and mortality [3, 7–9]. The SSC was designed to routinely check information before the critical stages of a surgery, reinforcing safety practices and facilitating communication among the surgical staff [7, 10]. This tool, completed in approximately 3 min, ensures essential verifications at three critical stages of surgery: before induction of anesthesia (Sign In), before skin incision (Time Out), and before the patient leaves the OR (Sign Out) [7]. It includes assessments such as confirming the patient’s identity, verifying the planned intervention, administering prophylactic antibiotics, performing sponge counts, and voicing concerns about the procedure [7].

Evidence indicates that increasing the reliability of surgical care processes can lead to considerable improvements [11–13]. Numerous studies have demonstrated that effective SSC implementation significantly reduces surgical complications and mortality rates [14–16]. In addition, it can improve communication and teamwork and reduce operating costs [17].

Despite its demonstrated benefits, the implementation of the SSC remains a challenge in many low‐ and middle‐income countries (LMICs) [18]. The limited resources, the inadequate awareness of its importance, and the persistent resistance to change are all factors that have contributed to suboptimal adherence [19, 20]. For instance, the WHO attempted a nationwide SSC implementation across 15 African countries in 2011. After one year, only one hospital had successfully implemented the checklist, and none of the countries had initiated a nationwide rollout [21]. A systematic review highlights that variability in SSC compliance across institutions and countries is often influenced by systemic, organizational, and human factors such as teamwork, leadership, and professional perceptions [22].

While surgeons, anesthetists, and nurses all have essential roles in the OR, the nursing perspective regarding the SSC is critical, as nurses are frequently tasked with championing it. Research has shown that nurses play a continuous and central role in patient advocacy and safety surveillance across all phases of a surgical procedure [23–25]. Yet, perioperative nurses face many challenges that limit their ability to ensure compliance, including complex power dynamics, workload, time constraints, and limited institutional support or formal authority [26].

In our context, the SSC was implemented in a Tunisian university hospital as part of a structured initiative involving staff training and support over a 15‐month period, followed by a four‐month follow‐up phase to promote sustainability. This process aimed to establish the institutional foundation for its regular use. However, the extent to which this implementation has been successfully integrated into routine practice and how it is perceived by perioperative nurses remain unclear. As nurses play a central role in ensuring SSC adherence and patient‐safety surveillance [27], understanding their experiences is essential to identify context‐specific barriers and facilitators, particularly in LMICs, where evidence remains limited.

Two earlier studies conducted in this same Tunisian context offer partial insight but leave this gap unaddressed. Prior work assessed OR professionals′ attitudes toward the SSC using a structured questionnaire, reporting generally favorable views but not examining actual compliance or qualitative experience [28]. More recently, perioperative safety perceptions and observed compliance with safety standards were examined across two Tunisian university hospitals, using direct procedure observation of 266 surgical procedures and a single‐item patient‐safety perception measure. However, that study addressed perioperative safety practices broadly rather than the SSC specifically and did not incorporate qualitative interviews [29]. Both studies were methodologically and analytically distinct from the present study, with separate data sources, instruments, and analyses; no data, transcripts, or findings were shared.

The present study builds on this body of research by addressing that gap directly and combining quantitative and qualitative approaches to capture both the extent of compliance and the perceptions of perioperative nurses, providing a more comprehensive interpretation of findings. Nevertheless, relatively few studies have integrated both strands through a mixed‐methods approach. Most studies examining the implementation of the WHO SSC have used either quantitative or qualitative approaches in isolation. Quantitative studies typically focus on measuring compliance rates, completion of checklist items, or associated patient outcomes, while qualitative research explores staff perceptions, attitudes, and contextual barriers to adoption.

This study aims to evaluate compliance with the SSC and explore the perceptions of perioperative nurses regarding its use. Using a mixed‐methods design, it assesses adherence rates through chart audit and investigates attitudes, barriers, and facilitators through interviews.

## 2. Methods

### 2.1. Study Design and Setting

This study used a sequential mixed‐methods design, in which quantitative data were collected followed by a qualitative phase, providing qualitative context to interpret the quantitative findings [30–32].

A retrospective review of patient medical records was conducted over a 19‐month period (comprising 15 months of intervention and 4 months of follow‐up) to assess SSC compliance and completion rates. Quantitative data were extracted at three time points across this period: midway through the intervention, immediately after full implementation, and after the follow‐up period.

This was complemented by qualitative interviews exploring perioperative nurses′ perceptions, experiences, and attitudes toward the SSC. Qualitative data collection lasted 3 months (from June to August 2021) and was carried out after the full‐scale implementation of the SSC in a tertiary hospital in the district of Sousse, Tunisia.

### 2.2. Quantitative Survey: SSC Compliance

#### 2.2.1. Sample Size and Sampling

To assess the compliance and completion of the SSC, we conducted a retrospective review of a randomly selected sample of medical records. To determine the minimum sample size required, we applied the sample size estimation method for descriptive cross‐sectional studies proposed by Kish (1965) [33].

To account for the wide range of reported compliance rates in the literature and the absence of a precise prior estimate in our setting, we set the compliance rate for our study at a conservative estimate of 50%. This approach ensured a representative sample of SSC compliance, enabling reliable analysis and comparisons.

Accordingly, the minimum sample size of medical records to withdraw from the archives was 600, which informed the sampling scheme that enabled a randomized selection of 40 medical records per month (10 per surgical specialty) over a 19‐month period (comprising 15 months of intervention and 4 months of follow‐up). Data collection was conducted across four OR archives, including thoracic and cardiovascular surgery, neurosurgery, orthopedic surgery, and reconstructive and plastic surgery.

All eligible surgical cases within each specialty were listed chronologically for every month, numbered, and then sampled using a systematic random approach by selecting every *n*th record until the monthly quota of 10 was reached. This equal‐allocation quota design yielded 10 records per specialty per month, giving equal representation to each of the four specialties irrespective of their underlying case volumes, ultimately resulting in a final sample of 760 medical records.

An illustrative example of the systematic sampling procedure applied for the first month of data collection across the four surgical specialties is presented in Table 1.

**TABLE 1:** Example of systematic sampling procedure for the first month across surgical specialties.

Specialty	Eligible cases for the first month of data collection	Sampling interval (*n*)	Records selected
Neurosurgery	73	7	10
Orthopedic	143	14	10
Thoracic/cardiovascular	101	10	10
Reconstructive/plastic	81	8	10

#### 2.2.2. Data Collection

Data collection required access to the hospital archives as medical records were not computerized. SSC forms were paper‐based and were stored within the patients’ medical charts.

Afterward, patients’ files were reviewed to assess SSC compliance and whether all three stages were appropriately completed. An Excel sheet was developed to record compliance and completion of each of its three components (Sign‐In, Time‐Out, Sign‐Out). The data collection sheet also included general indicators such as date of operation, type of surgery (emergency or elective), shift (day or night), type of anesthesia, and adherence to SSC items.

The study protocol was approved by the institutional ethics committee of the Faculty of Medicine of Sousse under the reference CEFMS 31/2019, and the study was conducted in accordance with the ethical principles outlined in the Declaration of Helsinki. Given the impracticability of tracking and contacting the large number of patients whose records were reviewed, individual patient consent was not sought for the retrospective chart review; this approach was specified in the approved protocol. Data extraction was limited to the date and type of operation and SSC compliance/completion, with no personal identifiers (such as patient name and address) collected. Authorizations to access the OR and the archives were obtained from all OR heads.

#### 2.2.3. Data Analysis

Collected data were entered into Microsoft Excel and exported into SPSS (Version 26) (IBM, SPSS Inc., Chicago, IL, USA). Data were analyzed descriptively using frequencies and percentages to assess overall SSC compliance and completion of individual items. Comparisons across specialties, surgery type, anesthesia type, and shifts were performed using chi‐square tests, with significance set at *p* < 0.05.

### 2.3. Qualitative Phase: OR Professionals’ Perspectives and Attitudes

#### 2.3.1. Participants

In line with the sequential mixed‐methods design, the subsequent part of the study consisted of semistructured interviews to explore perioperative nurses’ perceptions and attitudes toward the SSC. Participants were selected purposively using a critical case sampling approach in which individuals who were likely to provide the most information were recruited [31].

To ensure diversity, we included at least one participant from each OR specialty and from each professional role category (senior nurses, scrub nurses, circulating nurses, and OR nurse managers), all of whom had a background as OR nurses. Recruitment was facilitated with the assistance of the OR managers. Eligibility criteria required participants to have at least 2 years of experience in their respective OR and to be directly involved in SSC processes, thereby ensuring a deeper contextual understanding.

Interviews continued until data saturation was reached after 13 interviews, when no new themes emerged. The final sample was balanced across the different types of perioperative nurses at the hospital and included 2 senior nurses, 4 scrub nurses, 3 circulating nurses, and 4 OR nurse managers.

#### 2.3.2. Data Collection and Ethical Consideration

For the qualitative data collection, the research team developed a semistructured interview topic guide based on existing research on the SSC [34, 35] and tailored for the postintervention context of this study. The interview guide included eight open‐ended questions with optional probes, seeking to explore the participants’ perceptions of the SSC, barriers and facilitators to its use, the impact on teamwork and communication, and sustainability (Supporting File 1 outlines the interview topic guide used in the semistructured interviews). The topic guide was pilot‐tested for comprehension and clarity with a small number of OR professionals who did not take part in the actual data collection.

Before conducting the interviews, researchers first approached the participants, introducing themselves, describing their professional background, and explaining the study’s objectives and relevance. Afterward, interviews were scheduled according to participants’ availability and time preferences and were conducted in the OR manager’s office or another private space within the OR area, without the presence of anyone other than the interviewer and the participant. All interviews were conducted in the Tunisian/Arabic dialect, with durations ranging from 38 to 76 min, and none were repeated.

Two researchers (Wiem Aouicha and Mohamed Ayoub Tlili), both of whom have a professional background as OR nurses, conducted the interviews. Their familiarity with the OR context helped in the establishment of rapport, effective probing, and the appropriate interpretation of participants’ perceptions.

As for data capture, all interviews were audio‐recorded with the participants’ permission, and the recordings were transcribed verbatim. Subsequently, transcripts were returned to participants to verify their accuracy. The final transcripts were then translated into English by the principal investigator for analysis. To enhance translation accuracy and semantic equivalence, a second bilingual coauthor (Mohamed Ayoub Tlili), fluent in both Tunisian Arabic and English, independently reviewed the English translations against the original Arabic transcripts to verify that the meaning, tone, and contextual nuances were preserved. Any discrepancies were discussed and resolved collaboratively between the two researchers.

Participation was strictly voluntary, and informed consent was obtained from all the participants. The consent process explicitly informed participants that the study findings would be published and that anonymized quotes from the interviews, rather than complete transcripts, might be included in the resulting publications. They were also informed that they could decline participation or withdraw at any time without consequence. Anonymity and confidentiality were ensured by removing all personal identifiers from transcripts prior to analysis, and data were stored securely and used only for research purposes.

#### 2.3.3. Data Analysis

Thematic analysis was conducted on the English‐translated interview transcripts, following Braun and Clarke’s reflexive thematic analysis approach [36]. The analysis began with repeated readings of the transcripts to identify significant statements related to participants’ experiences and attitudes toward the SSC, which were then coded. Codes with similar meanings were grouped together and organized into multiple broader categories, which were then refined into five main themes through several iterative discussions within the research team [36].

To reduce the risk of bias and enhance trustworthiness, two researchers independently coded the transcripts. Coding decisions and interpretations were compared and discussed in multiple meetings. Any discrepancies were resolved through consensus within the research team [37]. Additionally, credibility was supported by sharing the preliminary findings with interviewees to confirm that their perspectives and meanings had been accurately represented.

## 3. Results

### 3.1. SSC Compliance Survey

#### 3.1.1. Sample Characteristics

A total of 760 surgical procedures were reviewed to assess the use of the SSC. The majority of procedures were elective (*n* = 592, 77.9%) and performed during the day shift (*n* = 615, 80.9%). General anesthesia was the most common anesthetic technique, administered in 492 cases (64.7%), followed by spinal anesthesia in 213 cases (28.1%). Cases across the four surgical specialties (thoracic/cardiovascular surgery, neurosurgery, orthopedic surgery, and reconstructive and plastic surgery) were evenly distributed following the medical records’ sampling method.

#### 3.1.2. SSC Compliance Over 19 Months

Of the 760 medical records reviewed, 302 included an SSC, corresponding to a compliance proportion of 39.7% within the study sample over the 19‐month period. Compliance increased during the intervention phase, reaching a peak of 60% after 8 months (Figure 1). The mean compliance during the 15 intervention months was 42.5%. During the 4‐month follow‐up period, compliance declined, averaging 29.4%.

**FIGURE 1:**
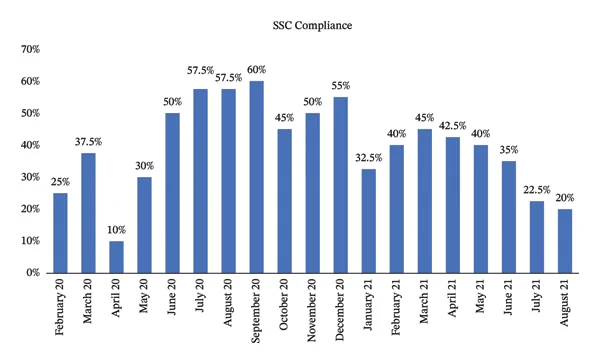
Distribution of the surgical safety checklist compliance over 19 months.

#### 3.1.3. Differences by Surgical Urgency, Anesthesia Type, Shift, and Surgical Discipline

SSC use was significantly higher in elective than in emergency procedures (*p* < 0.001) and during day shifts compared with night shifts (*p* < 0.001). In contrast, SSC use did not significantly differ by type of anesthesia (*p* = 0.877).

Most notably, SSC compliance varied across surgical disciplines, with neurosurgery showing the highest compliance rate (58.4%) and orthopedic surgery the lowest (22.6%), as shown in Table 2.

**TABLE 2:** Differences for urgency, type of anesthesia, shift, and surgical discipline.

Characteristics	SSC used	SSC not used	Within‐group SSC compliance rate	*p* value
Type of surgery				< 0.001**^1^**
Elective	285	307	48.1% (285/592)	
Emergency	17	151	10.1% (17/168)	
Shift				< 0.001**^1^**
Day	289	326	47.0% (289/615)	
Night	13	132	9.0% (13/145)	
Type of anesthesia				0.877^2^
General	197	295	40.0% (197/492)	
Other	105	163	39.2% (105/268)	
Surgical discipline				< 0.001**^1^**
Thoracic/cardiovascular	83	107	43.7% (83/190)	
Neurosurgery	111	79	58.4% (111/190)	
Orthopedic surgery	43	147	22.6% (43/190)	
Reconstructive/plastic	65	125	34.2% (65/190)	

*Note:* Bold values indicate statistically significant results (*p* < 0.05).

^1^ *p* values calculated using the chi‐square test.

^2^ *p* value calculated using Fisher’s exact test. Statistical significance set at *p* < 0.05.

#### 3.1.4. SSC Completion

Of the 302 SSCs retrieved, 196 (64.9%) were fully completed, with all items checked, while 106 (35.1%) were partially completed. Section‐level analysis showed variation in completion rates as presented in Figure 2. The Sign‐In section had the highest rate of completion (82.8%), followed by Time Out (72.2%) and Sign Out (64.9%). Notably, the Sign‐Out section was omitted in 19.2% of cases.

**FIGURE 2:**
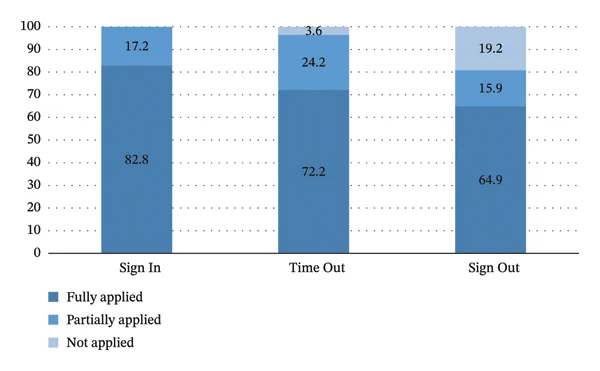
SSC completion for each section (*n* = 302). Bars showing the percentage of SSCs in which each section (Sign In, Time Out, and Sign Out) was fully applied, partially applied, or not applied.

#### 3.1.5. Missed Items Analysis

A total of 302 SSCs, each including 19 checklist items, resulted in 5738 potential checks, of which 633 (11.0%) were missed (Table 3). The Sign‐In section accounted for 18.2% of all missed items, whereas the Sign‐Out section accounted for the majority (54.8%). The most frequently missed item was Item 19 (“*Surgeon, nurse, and anesthesiologist review aloud the key concerns for the recovery and care of the patient*”), which was omitted 87 times (13.7% of all missed items). Other commonly missed elements included specimen labeling (Item 17, 10.9%) and equipment issue reporting (Item 18, 11.7%).

**TABLE 3:** Missing items in the collected surgical safety checklists (*n* = 302).

SSC section	Item no	SSC item	Number of times it was missed (n)	Percentage (%)
Sign In	1	Has the patient or his/her legal guardian confirmed his/her identity, site, procedure, and consent	7	1.1
	2	The surgical site is marked, or site marking is not applicable	37	5.8
	3	The anesthesia machine and medication checks are complete	14	2.2
	4	The pulse oximeter is on the patient and functioning	10	1.6
	5	All members of the team are aware of whether the patient has a known allergy	17	2.7
	6	The patient’s airway and risk of aspiration have been evaluated, and appropriate equipment and assistance are available	13	2.1
	7	If there is a risk of blood loss of at least 500 mL (or 7 mL/kg of body weight, in children), appropriate access and fluids are available	17	2.7
		Subtotal	115	18.2

Time Out	8	Confirms that all team members have been introduced by name and role	24	3.8
	9	Confirms the patient’s identity, surgical site, and procedure	14	2.2
	10	Confirms that prophylactic antibiotics have been administered ≤ 60 min before incision is made or that antibiotics are not indicated	21	3.3
	11	Surgeon reviews critical and unexpected steps, operative duration, and anticipated blood loss	54	8.5
	12	Anesthesiologists review concerns specific to the patient	31	4.9
	13	Nursing staff review confirmation of equipment availability and sterility, and other concerns	13	2.1
	14	Confirms that all essential imaging results for the correct patient are displayed in the operating room	14	2.2
		Subtotal	171	27.0

Sign Out	15	Name of the procedure as recorded	59	9.3
	16	That the needle, sponge, and instrument counts are complete (or not applicable)	58	9.2
	17	That the specimen (if any) is correctly labeled, including with the patient’s name	69	10.9
	18	Whether there are any issues with equipment to be addressed	74	11.7
	19	Surgeon, nurse, and anesthesiologist review aloud the key concerns for the recovery and care of the patient.	87	13.7
		Subtotal	347	54.8

		Total missed items	633	100

### 3.2. Qualitative Phase Results

Participants were aged 29 to 52 years, and most were women (8 out of 13). The majority of them had over 10 years of work experience (10/13) and more than 12 years of experience in their OR (9/13).

Analysis of the 13 interviews with perioperative nurses generated five main themes: (1) Shifting Attitudes: From Skepticism to Acceptance; (2) Barriers to SSC Compliance; (3) Facilitators of SSC Use; (4) Perceived Impact on Teamwork and Communication; and (5) Sustainability and Improvement Needs.

#### 3.2.1. Shifting Attitudes: From Skepticism to Acceptance

All participants acknowledged that the SSC enhanced patient safety and provided structure to perioperative processes. The majority of interviewees (10/13) highlighted a shift from skepticism to appreciation.

Scrub Nurse 1*: “At first, when we were informed about the SSC, a lot of colleagues thought it was just more paperwork*. *However, eventually I personally saw how useful it was in structuring the work and preventing mistakes. It makes everyone stop and think before continuing.”*

In most interviews (10/13), participants described the checklist as a “safety net” that safeguarded against omissions by reminding staff of essential steps, particularly during complex cases, at the start of surgery, or in situations with back‐to‐back procedures where the likelihood of errors was heightened.

Scrub Nurse 4*: “The checklist is a kind of safety net that nurses could depend on. Even if we are tired, it reminds us of details that can make a big difference for the patient.”*

One senior nurse described how a routine step in the checklist turned into a defining moment for the team’s perception of its value.

Senior Nurse 2: *“During Sign In, the circulating nurse asked the patient about allergies. The patient revealed a severe reaction to penicillin, that was not mentioned in the file, just as we were about to administer it. After that, the nurses’ insistence on the checklist made sense to everyone. It was truly protecting the patient.”*

Before its implementation, the use of the checklist was fragmented and dependent on individual initiative. Several participants (6/13) recalled that it was not formally embedded in hospital policy.

Senior Nurse 1: *“I tried to use it before, but it depended on the team I worked with during the surgery. There was no obligation, no official guidance.”*

OR Nurse Manager 4: *“It was more of a personal effort in the past. If you believed in it, you did it, if not, nobody said anything.”*

For some (3/13), the checklist remained more of a task than a genuine safety tool. In particular, nurses expressed frustration when their efforts to conduct a meaningful pause were met with disengagement.

Scrub Nurse 3: “*Sometimes we just tick the boxes quickly because the surgeon is impatient. It loses its purpose if I can’t get the team to genuinely engage.”*

However, most participants (8/13) emphasized the importance of genuine engagement while *running the SSC to guarantee its preventive value.*

Circulating Nurse 2: *“When the whole team is engaged in the process, running the checklist feels important. But, if nobody pays attention, it feels like I’m just talking to myself, and that’s when safety is compromised.*”

#### 3.2.2. Barriers to SSC Compliance

Several barriers were identified as limiting consistent SSC use. Time pressure, especially during emergencies, was the most frequently cited challenge by participants.

Circulating Nurse 3: *“During an emergency, honestly, we just think about saving the patient, and the checklist is usually forgotten.”*

In addition, participants (10/13) mentioned that staff shortages and workload pressures interfered with completion of the checklist.

OR Nurse Manager 1: *“There are days when we are short-staffed and overwhelmed. In those cases, the checklist is not the team’s priority.”*

Hierarchical structures were reported as a major barrier by all nursing staff participants. They felt responsible for initiating the SSC but often powerless to enforce its completion against surgical authority. Even when staff were aware of its significance, they felt hesitant to insist on its completion if the senior surgeon signaled impatience or resistance.

Scrub Nurse 2: “When the surgeon is in a hurry, no one wants to insist on completing the checklist.”

Junior nurses (*n* = 3), in particular, felt that speaking up could be perceived as challenging authority.

Scrub Nurse 2: “As a junior, I don’t want to look like I am correcting my senior. So even if I notice another nurse is trying to run the checklist and it’s being ignored, I just stay quiet.”

Some participants reflected that these barriers were particularly pronounced in a hospital where the SSC was newly introduced and not yet embedded in daily routines.

Senior Nurse 1: *“Because it was never part of our system before, people still see it as an extra work, not as part of the surgery itself.”*

In addition, participants (8/13) pointed out that running the SSC in a distraction‐prone environment was another challenge frequently observed. With multiple simultaneous tasks occurring at once, running the checklist items sometimes interfered with ongoing surgical routines, which often led to incomplete or rushed execution.

Circulating Nurse 1: “*Sometimes the checklist is started but then interrupted. By the time we continue, people have already lost interest and moved on.”*

Scrub Nurse 2: “*We always begin with the checklist, but by the end of surgery everyone gets preoccupied; packing instruments, waking the patient, writing notes. The Sign Out is very often skipped*.”

Another barrier mentioned by participants (4/13) was the lack of accountability mechanisms and follow‐up, which weakened compliance. Without visible consequences for neglect, many felt that the checklist was easy to bypass.

OR Nurse Manager 4: “*Nobody checks if we do it or not. So, if the team decides to skip, nothing happens.*”

Several participants (5/13) linked this lack of ownership to the SSC’s novelty and absence from hospital policy.

#### 3.2.3. Facilitators of SSC Use

Regarding factors that facilitated SSC use, strong leadership and active involvement were described as the key enablers. In fact, most of the participants (11/13) highlighted that when senior surgeons visibly endorsed and led the checklist, compliance improved noticeably. All interviewed nurses noted that they felt empowered to lead the checklist more effectively when the surgeon expressed interest.

Scrub Nurse 2: “*When the surgeon openly encourages the checklist, everyone engages, and we all share responsibility for patient safety. As a nurse, I feel empowered because it’s no longer just my job*.”

All the interviewed nurses described the implementation of the checklist as a turning point, since the SSC had never been previously institutionalized in the hospital and relied instead on informal, individual‐driven effort.

Senior Nurse 2: “*Before, it depended on who cared about it. After the implementation, it became part of the surgery, not just a personal choice*.”

The implementation strategy introduced structured training sessions, posters, and external follow‐up to standardize and support checklist use, giving it legitimacy and encouraging staff engagement.

OR Nurse Manager 4: “*The project gave us the push we needed. With the posters, the sessions, and people reminding us, it became more natural to use it*.”

Scrub Nurse 1: “*When the research team came and trained us, it was the first time we saw it as something official and not just optional.”*

Another key facilitator mentioned by participants (5/13) was peer support and mutual reminders, which helped normalize SSC use even when formal leadership was inconsistent. Encouragement from colleagues created a sense of accountability within the team.

OR Nurse Manager 3: “*As the OR manager, I take it upon myself to visit each operating room before the surgery and remind the team, ‘don’t forget the checklist.’ That helps maintain a sense of ownership*.”

According to two OR nurse managers, a pivotal facilitating factor was the emergence of nursing champions in the OR who actively engaged in the process and took personal ownership of the SSC’s success.

OR Nurse Manager 1: “*We had a few nurses who really became the checklist’s biggest supporters. They would gently remind their colleagues, ′Let’s do our safety check,′ and show how to do it quickly but thoroughly. Their enthusiasm was contagious*.”

#### 3.2.4. Perceived Impact on Teamwork and Communication

Most participants (11/13) perceived the SSC as a tool that fostered collaboration and open communication in the OR. They reported that the checklist helped shift the culture from task‐focused individual work to a shared responsibility.

Scrub Nurse 1: “*Before, everyone focused only on their own task. Now, during the checklist, we talk together. It creates unity*.”

Many participants (11/13) also perceived that the checklist improved efficiency and coordination within the team, describing how the SSC reduced misunderstandings and prevented redundant work by ensuring a shared understanding among team members.

OR Nurse Manager 3: “*Once we go through the checklist, we know exactly what each person is doing before the real work starts. It makes everything flow smoother*.”

Scrub Nurse 1: “*The OR is usually busy, so the checklist brings a moment where we all breathe, check, and make sure nothing is missed. It makes teamwork feel natural.”*

Participants (7/13) perceived that the structured format of the SSC created intentional pauses for dialog, which made room for team members to share critical information and voice their concerns.

Scrub Nurse 2: “*When something is up, I feel more comfortable sharing it because the checklist gives me the opportunity.”*

Scrub Nurse 4: “*The checklist makes the environment more open to speaking up, because everyone is expected to listen at that time.”*

#### 3.2.5. Sustainability and Improvement Needs

Although participants generally acknowledged the value of the SSC, many (8/13) expressed uncertainty about its long‐term sustainability once the initial momentum of the project declined. A recurring concern was that, without reinforcement and oversight, old practices could easily resurface.

OR Nurse Manager 3: “*I fear that we will go back to old habits without follow-up*.”

Almost all participants (12/13) emphasized the need for continuous education to sustain compliance. Initial training was viewed as useful, and refresher sessions and practical coaching were recommended.

Circulating Nurse 1: “*I feel that regular refresher training and ongoing encouragement are mandatory. Otherwise, people will stop using it*.”

Participants (6/13) repeatedly stressed the importance of embedding the SSC within hospital policies and regulations.

OR Nurse Manager 1: “*It has to become part of the hospital rules, that will make it not optional. We need it to become official.*”

Sustained compliance, according to some interviewees (5/13), depended on systematic monitoring and constructive feedback. Regular audits and public recognition of complying teams were suggested as strategies to support continued use.

OR Nurse Manager 4: “*If no one checks, people will stop. But if there is feedback, it reminds us that it matters*.”

Scrub Nurse 3: “*Recognition also helps. When complying teams see that their efforts are valued, they become more motivated to continue.”*

Another sustainability strategy suggested by participants (5/13) related to the empowerment and formal recognition of nursing champions. They highlighted that having motivated personnel, particularly nurses, in each OR was essential to serve as local advocates and leaders.

OR Nurse Manager 2: “*We need to officially recognize checklist champions in each OR and give them the authority to mentor others, audit compliance, and provide coaching, when needed. Having these champions will definitely improve sustainability*.”

Finally, participants (3/13) suggested that digitalizing the SSC and integrating it into the hospital’s electronic records could improve usability and accountability, because unlike paper forms, a digital form would be harder to ignore and could provide real‐time tracking.

Senior Nurse 1: “*If it was in the computer, linked to the patient file, it would be harder to ignore. On paper, it feels optional*.”

### 3.3. Findings Integration

Table 4 presents the integrated quantitative and qualitative findings and highlights areas of complementarity and difference. Overall, the findings were broadly complementary, but the correspondence between qualitative themes and quantitative patterns should be interpreted cautiously when the relevant constructs were not directly measured. In particular, leadership, teamwork, hierarchy, and acceptance were explored qualitatively and should therefore be regarded as contextual explanations rather than evidence of quantitative convergence.

**TABLE 4:** Integrated summary of qualitative themes, subthemes, and key codes with corresponding SSC audit findings.

Main theme	Subthemes	Main codes/key ideas	Quantitative SSC audit findings	Qualitative–quantitative correspondence
1. Shifting Attitudes: From Skepticism to Acceptance	• Initial skepticism and resistance • Checklist value for patient safety • Variation in engagement • Importance of genuine engagement	‐ Perceived as extra paperwork at first ‐ Gradual appreciation after perceiving safety benefits ‐ Perceived to prevent omissions and errors ‐ Dependent on team culture and leadership ‐ Checklist seen as meaningful when all team members engage in it ‐ Superficial or rushed completion	‐ SSC compliance proportion within the study sample: 39.7% (302/760); ‐ Peak during intervention: 60% ‐ Mean intervention compliance: 42.5%; ‐ Follow‐up compliance decline: 29.4%; ‐ Full completion rate: 64.9%	Descriptive pattern: The rise in compliance during the intervention phase occurred alongside qualitative reports of improved acceptance. This correspondence is descriptive and does not establish that the intervention caused the observed change in compliance.

2. Barriers to SSC Compliance	• Time pressure and workload • Staff shortage • Hierarchical culture • Distraction and competing priorities • Lack of accountability and policy support	‐ Emergencies limit the application of the checklist ‐ Workload limits compliance ‐ Power imbalance: nurses hesitant before surgeons ‐ Junior nurses fear challenging authority ‐ Checklist is seen as “extra” not integrated ‐ Interruptions in the OR lead to incomplete checklists ‐ Sign Out phase frequently overlooked ‐ No monitoring or consequences for not using the SSC ‐ Absence of formal hospital policy	‐ SSC used in elective vs emergency: 94.4% vs 5.6% of used SSCs ‐ Significant difference in SSC compliance between elective and emergency procedures (*p* < 0.001); ‐ Sign Out most missed section (54.8% of all omissions); ‐ Item 19 was most frequently missed (13.7%)	Complementary (qualitative explanation): Emergency context was directly reflected in the audit data through the significantly lower compliance in emergency cases, whereas workload, hierarchical culture, and accountability were identified qualitatively and were not independently measured in the audit. Hierarchical culture may provide qualitative context for patterns of low compliance and frequent Sign‐Out omissions, but this relationship was not directly measured.

3. Facilitators of SSC Use	• Leadership and surgeon engagement • Institutionalization and formal training • Visual reminders and follow‐up • Peer support and teamwork • Role of nursing champions	‐ Senior surgeon endorsement was perceived to support compliance ‐ Nurses described feeling more empowered when leadership supported checklist use. ‐ Training sessions and posters increased awareness ‐ Peer encouragement created accountability ‐ Nursing champions promoted consistency and motivation	‐ Higher SSC use in day shift (95.7% of SSCs used in day vs night 4.3%); ‐ Neurosurgery highest contribution to SSC completion (36.8%)	Complementary (qualitative explanation): Day shift and neurosurgery showed higher compliance in the audit data; qualitative themes of leadership presence and structured environments offer a plausible explanation for this pattern, but leadership, teamwork, and the degree of institutional support were not directly measured quantitatively.

4. Perceived Impact on Teamwork and Communication	• Shared responsibility and collaboration • Improved coordination and workflow • Enhanced communication and speaking up culture	‐ Shift from individual to team focus ‐ Clearer roles and reduced misunderstandings ‐ Encourages joint reflection before procedures ‐ Promotes mutual respect and open dialog ‐ Creates structured moments for information exchange ‐ Increased comfort in voicing safety concerns	‐ Sign In highest completion (82.8%), followed by Time Out (72.2%) and Sign Out (64.9%); ‐ 35.1% of SSCs partially completed	Complementary (qualitative explanation): Participants perceived improved communication and teamwork in relation to checklist use; the quantitative data reflect only checklist‐phase completion rates (Sign In, Time Out, Sign Out), which were not designed to directly measure teamwork or communication. The comparatively lower completion of the Sign Out phase indicates variability in checklist adherence toward the end of the surgical procedure but does not, by itself, demonstrate changes in team engagement.

5. Sustainability and Improvement Needs	• Risk of regression postimplementation • Need for continuous training and feedback • Policy integration and monitoring • Recognition and motivation • Empowering SSC champions • Digitalization for accountability	‐ Concern about losing momentum without follow‐up ‐ Refresher sessions and continuous education needed ‐ Integration into hospital policy for long‐term compliance ‐ Regular audits and feedback improve motivation ‐ Recognition of compliant teams to boost morale ‐ Appointing OR “checklist champions” ‐Digital SSC could enhance tracking and accountability	‐ Overall decline in compliance after intervention (42.5% ⟶ 29.4%); ‐ 35.1% partially completed SSCs; ‐ 19.2% omission of Sign‐Out phase	Descriptive pattern: The observed decline in compliance after the intervention phase occurred alongside participants’ concerns about losing momentum without follow‐up, illustrating a sustainability challenge. This pattern should be interpreted descriptively and does not demonstrate a causal effect of ending or reducing the intervention.

The rise in SSC compliance during the intervention phase occurred alongside participants′ accounts of shifting from initial skepticism to gradual acceptance. This correspondence is descriptive: It does not establish that the intervention caused the observed change in compliance.

Similarly, the quantitative audit showed substantial variation in SSC compliance across clinical contexts, particularly between elective and emergency procedures and across shifts and specialties. Qualitative findings provided contextual insight into these patterns, with nurses describing time pressure, workload, and emergency situations as barriers, while leadership support, structured work environments, teamwork, and peer support were described as facilitators. Of these factors, the lower compliance observed in emergency procedures was directly demonstrated by the quantitative audit. In contrast, leadership, hierarchical culture, teamwork, and accountability were not independently measured in the audit, and therefore, their associations with compliance cannot be quantitatively confirmed.

Regarding teamwork and communication, nurses perceived the SSC as supporting communication, collaboration, coordination, and speaking up within the OR. The quantitative audit, however, assessed checklist completion rather than teamwork or communication directly. Although Sign In and Time Out had higher completion than Sign Out, the lower completion of Sign Out indicates variability in adherence to the checklist process but does not demonstrate changes in team engagement or communication. Thus, the qualitative findings add contextual meaning to the potential team‐related benefits of SSC use without providing direct quantitative confirmation.

Finally, the quantitative findings showed a descriptive decline in SSC compliance from the intervention phase to follow‐up (42.5% to 29.4%). This pattern occurred alongside qualitative concerns about sustaining momentum once formal implementation support ended. Together, these findings point to a sustainability challenge: The audit demonstrates a measurable drop in compliance, while participants′ accounts highlight the need for ongoing reinforcement to maintain SSC use over time. Nevertheless, this trend cannot be directly attributed to the reduction or discontinuation of the intervention, as other factors may also have contributed.

## 4. Discussion

Our study evaluated compliance and completion of the WHO SSC postimplementation in a Tunisian university‐hospital setting, and integrated quantitative assessment with qualitative insights from perioperative nurses with regard to their attitudes and perceptions of this tool. While high‐income countries (HICs) report relatively high compliance and improved outcomes [14, 38, 39], evidence from LMICs remains limited [40–42]. By reporting compliance patterns alongside the nurses’ perceptions, our mixed‐methods study contributes to closing this evidence gap.

Overall, the qualitative and quantitative findings were largely complementary: Qualitative insights offered plausible explanations for the quantitative patterns in compliance, variation across surgical phases, and differences according to clinical context, although constructs such as leadership, teamwork, hierarchy, and acceptance were explored only qualitatively and were not independently quantified by shift or specialty.

### 4.1. Compliance and Completion of the SSC

Compliance with SSCs varies across contexts and is influenced by multiple factors [14]. A global study by Delisle et al. [39] across 94 countries reported that the overall SSC use was 75%. Subgroup analysis revealed that rates exceeded 85% in HICs but remained below 30% in LMICs [39]. These disparities reflect substantial variations in resources, monitoring systems, and local leadership between the two contexts [39, 43–45].

In our study, SSC compliance was, on average, 42.5% over 15 months, peaking at 60% after 8 months and then declining to 29.4%. This pattern of an initial peak followed by a decline was reported in several studies where overall compliance fluctuated, with peak compliance levels observed at the beginning [46, 47]. In addition, this pattern is consistent with interviewees’ statements that compliance tends to peak alongside “*structured training sessions, posters, and external follow-up*” provided at the beginning of the implementation, offering a plausible explanation for the trend observed, though a causal effect cannot be confirmed without a control group. Additionally, the later compliance drop mirrors the staff’s concern about sustainability, captured in this quote, “*I fear that we will go back to old habits without follow-up*.” This phenomenon, often called “initiative decay,” is common in LMIC settings where project‐based implementations fail to move to sustained, funded programs, resulting in a return to previous practices [44, 45]. A recent literature review by Eco et al. highlighted that the major challenges in quality improvement endeavors are the need for adequate funding, sufficient personnel training, and appropriate systems for consistent data collection [48].

Of the 302 SSCs reviewed, 196 were fully completed, making the completion rate 64.9%. The Sign‐In section was applied (82.8%) in most cases, which is consistent with studies from Switzerland (93%) [38], Ethiopia (83%) [49], and New Zealand (69%) [50]. The placement of the Sign‐In section at the beginning of surgery, when OR personnel are vigilant and attentive to guarantee a smooth transition from preparation to procedure, accounts for its higher completion rate [51].

On the other hand, our quantitative data revealed a critical gap: More than half of all missing items were in the Sign‐Out area, which was the most commonly overlooked. The qualitative data support this finding and identify the reasons for it. Participants described a “distraction‐prone environment” where, by the end of surgery, “*everyone gets preoccupied; packing instruments, waking the patient, writing notes. The Sign Out is very often skipped*.” This evidence reveals a consistent pattern that has been well documented in the literature [52–54]: The final safety verification, or “Sign Out,” is routinely compromised by fragmented procedures and conflicting tasks. A recent literature review and meta‐analysis by Habtie et al. showed that the Sign‐Out section remained poorly addressed because it is extremely vulnerable to disruptions and team dispersal even in high‐compliance settings, seriously impairing its safety function [55].

### 4.2. Barriers and Facilitators

Time pressure and high workload negatively influenced checklist use, particularly in emergencies. Compliance was higher in elective surgeries than in urgent cases, reflecting other research findings where SSC use is lower in urgent procedures (odds ratio 0.68, 0.53–0.86) [39]. The quantitative result indicating markedly reduced compliance during emergencies (*p* < 0.001) is articulated by our participants’ quotes: “*During an emergency, honestly, we just think about saving the patient, and the checklist is usually forgotten*.” Hesitancy in urgent scenarios also stems from concerns that checklist use might delay lifesaving procedures or increase postoperative risks [39, 45, 56]. Yet, evidence shows SSC implementation in urgent noncardiac surgeries can reduce complications by over one‐third [57–59]. Condensed checklists focusing on essential safety items have been suggested to address time pressures in urgent cases [38].

On the other hand, considerably poorer SSC compliance was recorded during night shifts (*p* < 0.001). This discrepancy may be a result of power dynamics and hierarchical culture within the OR rather than just timing. Our interviewed nurses felt that they were responsible for initiating the SSC but often felt powerless to enforce its completion against surgical authority.

Senior surgeons’ absence exacerbates this power disparity by depriving nurses of the top‐down support required to advocate for the checklist procedure. This phenomenon is supported by an ethnographic study that found that without explicit leadership support, nurses’ authority to enforce safety protocols remains weak, particularly during off‐hours, creating a critical safety gap [60]. The same study also highlights a concerning paradox: Whereas perioperative nurses place a high value on the SSC’s contribution to patient safety, their own position in the multidisciplinary team is frequently limited [60]. This finding is relevant since a nurse’s perceived authority directly affects their capacity to promote safety protocols [61]. Positive surgical leadership clearly has a substantial effect. As one scrub nurse described, she felt empowered to initiate the checklist when the surgeon visibly endorsed it. Strategies aimed at improving communication between medical and nursing staff by creating a culture of safety may help nurses engage more effectively with the multidisciplinary team to conduct the SSC [60].

Despite these challenges, several facilitators emerged. Educational support was institutionalized through formal training sessions and visual reminders, which systematically increased awareness and reinforced the SSC as a standard of care. Peer reinforcement through daily reminders also helped integrate the checklist into routine practices.

### 4.3. Perceived Impact on Teamwork, Communication, and Sustainability

Beyond compliance, participants perceived that the SSC positively influenced team dynamics. They reported that it created structured opportunities for dialog, reduced misunderstandings, and promoted a culture of shared responsibility. As a scrub nurse noted, “*Before, everyone focused only on their own task. Now, during the checklist, we talk together. It creates unity*.”

The integration of declining compliance data with qualitative findings highlights sustainability challenges. Participants recommended continuous education, systematic monitoring, constructive feedback, and formal integration into hospital policies. Digitalizing the SSC was also suggested to improve accountability and acceptance. Research has shown that SSC completion rates significantly increased with the use of electronic checklists [62, 63].

To ensure the SSC’s long‐term sustainability, healthcare institutions should explicitly integrate a program of safety culture champions into their implementation approach [64]. This is supported by a substantial body of implementation research and also the interviewees’ recommendations to sustain SSC use in the OR. In fact, nurse champions have proven to be an important component for successfully implementing new procedures and technology, both within and outside of healthcare settings [65, 66]. The role of these nurse champions extends beyond simply reminding their peers; they are essential in coaching them on both the technical aspects of processes and safety culture principles, thereby bridging the gap between policy and practice. Furthermore, research specific to the SSC confirms that in environments where the checklist was successfully implemented, the use of designated checklist champions was a key facilitating strategy [64]. To be effective, this approach must be actively supported by organizational actions, such as providing dedicated time and authority. Its impact also should be monitored through regular surveys and ongoing communication that consistently emphasize that “*patient safety is our number one priority*” [67].

### 4.4. Limitations

This study provides valuable insights about the SSC implementation in LMICs, although some limitations should be acknowledged. First, data were obtained from a single hospital, which may limit generalizability; however, the findings may be transferable to similar resource‐limited contexts. Second, quantitative data were collected from medical records, which may not entirely capture real‐time checklist use but may reflect routine clinical practice. Additionally, because compliance was assessed through sequential chart‐audit snapshots without a concurrent control group, the observed intervention‐to‐follow‐up pattern is best interpreted descriptively rather than as confirmed evidence of a causal intervention effect.

The equal‐allocation sampling design (10 records per specialty per month) deliberately gave equal weight to each of the four surgical specialties regardless of their underlying case volumes. Consequently, the overall compliance rate should not be interpreted as a hospital‐wide estimate of SSC use. Comparisons by urgency, shift, and anesthesia type should also be interpreted with this limitation in mind.

On the qualitative side, participants′ reports of improved teamwork, efficiency, and error prevention reflect subjective perceptions gathered through interviews; these constructs were not directly measured by the quantitative audit, which assessed only checklist‐phase completion and compliance rates.

The qualitative component drew on a purposive sample of perioperative nurses, providing in‐depth but context‐specific insights into the nurses’ experiences regarding the SSC that may not extend to other OR professionals or institutions.

Finally, the study did not directly assess patient outcomes; however, the combination of quantitative and qualitative methodologies increases the validity of our conclusions.

### 4.5. Implications for Nursing Management

Translating these findings into practice calls for action across several areas. First, SSC use should be integrated into standard operating procedures and monitored as a routine quality indicator rather than relying on staff goodwill or a time‐limited project. This may help prevent the “initiative decay” reported in similar LMIC settings [44, 45, 48]. Nursing champions should be formally designated, with dedicated time and clear responsibility to support colleagues, monitor compliance, and report recurring omissions to leadership. These conditions have been associated with effective champion‐led implementation in other settings [64–66]. Psychological safety should also be supported in daily OR practice through clear and practical behaviors [68]. These include senior surgeons actively encouraging questions during Time Out, explicitly supporting staff who pause the procedure when a safety step is missed, and prioritizing nonpunitive debriefs when checklist steps are skipped. A supportive team climate has recently been linked to measurable patient‐safety outcomes, particularly in nursing‐dominated teams [69].

Compliance monitoring should move from random audits to regular audit‐and‐feedback cycles, with results reviewed over time and reported by shift and specialty. This would allow managers to identify and address areas with lower compliance, such as night shifts or specific specialties. Regular audit and feedback, particularly when associated with a clear improvement plan, remains an effective approach for improving clinical practice [70]. Finally, training should be ongoing rather than limited to a single session, with particular attention to new staff and units with lower compliance [62, 63]. For emergency or high‐pressure cases, a shorter version of the checklist focusing on essential safety items may help maintain key safety checks without causing unacceptable delays [38].

## 5. Conclusions

This mixed‐methods study suggests that the SSC’s long‐term success depends less on its design than on the organizational culture in which it is implemented. The reported decrease in compliance postimplementation and the difficulties faced by nurses, particularly the hierarchical power dynamics that leave them feeling responsible for the SSC yet powerless to enforce it, suggest that the checklist reflects preexisting systemic problems. Addressing these problems requires organizational changes and, more fundamentally, a shift in perspective: from viewing the SSC as a project‐based initiative or a technical tool, to embracing it as a catalyst for building a collaborative and accountable surgical environment that prioritizes patient safety.

**Nomenclature**
HICs: High‐income countries
LMICs: Low‐ and middle‐income countries
OR: Operating room
SSC: Surgical safety checklist
WHO: World Health Organization

## Author Contributions

Conceptualization: Wiem Aouicha and Mohamed Ayoub Tlili.

Methodology: Wiem Aouicha, Mohamed Ayoub Tlili, and Nick Sevdalis.

Investigation: Wiem Aouicha and Mohamed Ayoub Tlili.

Project administration: Wiem Aouicha.

Data curation and formal analysis: Wiem Aouicha and Mohamed Ayoub Tlili.

Writing–original draft: Wiem Aouicha.

Writing–review and editing: Wiem Aouicha, Mohamed Ayoub Tlili, Bushra Alshammari, Hamdi Lamine, Nojoud Abdullah Alrashidi, Nahed Mousa Saber, Amany Ali Abd Elsalam, Laila A. Hamed, Nick Sevdalis, Nadia Falah Shatti Al Enezi, Hind Mobark Fahd Alshamry, Maha Sanat Alrashedi, and Aziza Zakaria Faramawy Ali.

Supervision: Wiem Aouicha and Mohamed Ayoub Tlili.

## Funding

Funding from the Scientific Research Deanship at the University of Ha’il, Saudi Arabia, was received in November 2024 to support the completion of data analysis, manuscript preparation, and publication of this article.

## Conflicts of Interest

NS is the director of London Safety and Training Solutions Ltd., which offers training and improvement and implementation solutions to healthcare organizations and the pharmaceutical industry. The other authors declare no conflicts of interest.

## Supporting Information

Additional supporting information can be found online in the Supporting Information section.

## Supporting information


**Supporting Information** Supporting File 1 contains the interview topic guide for the semistructured interviews, including the key questions and probing prompts used to explore participants’ experiences and attitudes regarding the SSC implementation.

## Data Availability

The datasets used and/or analyzed during the current study are available from the corresponding author upon reasonable request.
