# Supplementary material for: Surgical Safety Checklist Implementation in a Tunisian University Hospital: A Mixed‐Methods Study of Compliance and Nurses’ Perspectives on Barriers, Facilitators, and Sustainability
Source: J Nurs Manag. 2026 Oct 20;2026:7712576. doi: 10.1155/jonm/7712576 (PMC13681348; doi:10.1155/jonm/7712576)
Supplement: Supplementary file 1 [file JONM-2026-7712576-s001.docx]

The interview topic guide used to conduct the semi-structured interviews is included as *Supplementary File 1*. This file contains the key questions and probing prompts that structured the qualitative data collection process.

**Interview Topic Guide:**

**1.*What are your overall thoughts about the SSC after the intervention?***

- **Probes:**
  - How did you feel about the SSC before compared to now?
  - Do you think it adds value to patient safety? Why or why not?

**2. *Can you tell me about your experience with the recent intervention to implement the SSC?***

- **Probes:**
  - Were there parts that you found less effective?
  - Did the intervention change your attitude or behavior regarding SSC use?

**3. *What difficulties or obstacles do you still face in using the SSC consistently?***

- **Probes:**
  - Can you describe situations when the checklist is skipped or rushed?
  - What prevents staff from completing it properly?
  - Are there cultural, organizational, or practical barriers?

**4. *What helps you and your colleagues use the SSC more effectively?***

- **Probes:**
  - Are there supportive practices that encourage compliance?
  - How do colleagues or leaders support you in using it?

**5. *How has the SSC influenced teamwork in the operating room since the intervention?***

- **Probes:**
  - Have you noticed improvements in how team members interact?
  - Do you feel more comfortable speaking up during checklist use?
  - Is communication more structured, or does it feel like a formality?

**7. *Who usually leads the SSC process in your OR, and how does this affect compliance?***

- **Probes:**
  - Does leadership style make a difference in checklist use?
  - Do you feel supported by senior staff when using the SSC?

**8. *Do you think SSC use will continue at the same level now that the intervention is over?***

- **Probes:**
  - What makes you optimistic or doubtful about sustainability?
  - What additional changes would help ensure the checklist is used consistently?
